# Hypoglycemic Potential of Aqueous Extract of *Moringa oleifera* Leaf and *In Vivo* GC-MS Metabolomics

**DOI:** 10.3389/fphar.2017.00577

**Published:** 2017-09-12

**Authors:** Washim Khan, Rabea Parveen, Karishma Chester, Shabana Parveen, Sayeed Ahmad

**Affiliations:** ^1^Bioactive Natural Product Laboratory, Department of Pharmacognosy and Phytochemistry, Faculty of Pharmacy, Jamia Hamdard New Delhi, India; ^2^Department of Pharmacy, Banasthali University Tonk, India; ^3^Department of Bioscience, Jamia Millia Islamia New Delhi, India

**Keywords:** drumstick, HPLC, GC-MS, HFD/STZ, pattern recognition

## Abstract

*Moringa oleifera* Lam. (family; Moringaceae), commonly known as drumstick, have been used for centuries as a part of the Ayurvedic system for several diseases without having any scientific data. Demineralized water was used to prepare aqueous extract by maceration for 24 h and complete metabolic profiling was performed using GC-MS and HPLC. Hypoglycemic properties of extract have been tested on carbohydrate digesting enzyme activity, yeast cell uptake, muscle glucose uptake, and intestinal glucose absorption. Type 2 diabetes was induced by feeding high-fat diet (HFD) for 8 weeks and a single injection of streptozotocin (STZ, 45 mg/kg body weight, intraperitoneally) was used for the induction of type 1 diabetes. Aqueous extract of *M. oleifera* leaf was given orally at a dose of 100 mg/kg to STZ-induced rats and 200 mg/kg in HFD mice for 3 weeks after diabetes induction. Aqueous extract remarkably inhibited the activity of α-amylase and α-glucosidase and it displayed improved antioxidant capacity, glucose tolerance and rate of glucose uptake in yeast cell. In STZ-induced diabetic rats, it produces a maximum fall up to 47.86% in acute effect whereas, in chronic effect, it was 44.5% as compared to control. The fasting blood glucose, lipid profile, liver marker enzyme level were significantly (*p* < 0.05) restored in both HFD and STZ experimental model. Multivariate principal component analysis on polar and lipophilic metabolites revealed clear distinctions in the metabolite pattern in extract and in blood after its oral administration. Thus, the aqueous extract can be used as phytopharmaceuticals for the management of diabetes by using as adjuvants or alone.

## Introduction

Changes in lifestyles reduced physical activity, increased obesity, and rapid changes in environmental complications contribute to the increasing the number of eventuality of diabetes mellitus (Shaw et al., 2010). Globally, an estimated 422 million adults were living with diabetes in 2014, compared to 108 million in 1980, rising from 4.7 to 8.5% in the adult population. WHO projects that diabetes will be the seventh leading cause of death in 2030 (World Health Organization, 2016). However, this figure was already reached by 2011 according to the International Diabetes Federation (IDF; Wild et al., 2004). Diabetes is a disease, which has surmise foremost public health importance because of its multimode complications. Among other metabolic disorders, our society wishes to have an urgent solution for diabetes mellitus. Though a number of studies have been carried out for the discovery of medicine from plant origin, still it needs large improvement.

Various types of plants have been used for several centuries worldwide not only as dietary supplements but also as traditional treatment regimens for many diseases. So far, a large number of traditionally claimed plant medicine has been tested for diabetes and some of them showed a promising therapeutic potential. Among these plants, a rapidly growing perennial tree, *Moringa oleifera* Lam. (belonging to family Moringaceae) is widely distributed in Asia, America, and Africa (Fahey and Sc, 2005). This plant has been used by ancient Romans, Greeks, and Egyptians. In Indian ethnotherapeutic system of medicine, *M. oleifera* is reported to possess hypoglycemic activity. Since it is a significant source of fats, proteins, beta-carotene, vitamin C, iron, potassium, and other nutrients (Mahmood et al., 2010). The WHO has recommended to *Moringa* as an alternative to imported food supplies for the treatment of malnutrition. Besides being edible, all parts of the *M. oleifera* have long been deployed for the treatment of numberless diseases, and for that reason, in many instances, it is been called as “Miracle Tree” (Mbikay, 2012). Now a days, some parts of this plant have drawn much attention and have been studied for its various biological activities, including antiatherosclerotic (Chumark et al., 2008), immune boosting (Miyoshi et al., 2004), cardiovascular diseases, antiviral (Murakami et al., 1998), antioxidant, antimicrobial, anti-inflammatory (Kumar Gupta et al., 2013) properties, and tumor suppressive effects in skin papilloma genesis, hepatocellular carcinoma cancer, colon cancer, and myeloma (Brunelli et al., 2010). Leaves of *M. oleifera* have been used as antiulcer, diuretic, anti-inflammatory, wound healing (Farooq et al., 2012), antifungal activity (Chuang et al., 2007), potent CNS depressant action (Pal et al., 1995), and antifertility activity (Tahiliani and Kar, 2000). Major phytoconstituents present in *M. oleifera* leaf are niazinin, niazimicin, β-sitosterol, glucomoringin, *n*-benzyl thiocarbamates, kaempferol, other natural antioxidant molecules including vitamins, minerals, and carotenoids (Anwar et al., 2007; Jaiswal et al., 2009; Dawood and Fathy, 2014).

Recently, some studies have been reported for the antidiabetic potential of *M. oleifera* leaves (Jaiswal et al., 2009; Dawood and Fathy, 2014), pods (Une Hemant et al., 2014), and seeds (Al-Malki and El Rabey, 2015), however, most of them were focused on leaf (Jaiswal et al., 2009; Divi et al., 2012; Gupta et al., 2012; Edoga et al., 2013; Efiong et al., 2013; Dawood and Fathy, 2014). Nevertheless, the majority of studies of the leaf has been done on alcoholic and its hydroalcoholic extract (Gupta et al., 2012; Efiong et al., 2013), but as per traditional claim, the aqueous extract is preferred for therapeutic purposes. Similar results were obtained on the aqueous extract of leaves which has already been proven for its safety after its oral administration (Awodele et al., 2012). In spite of several studies, still, a scientific study needs to be performed to prove its traditional claim regarding its antidiabetic potential. It was, therefore, thought worthwhile to evaluate the antidiabetic potential of *M. oleifera* leaf with a complete HPLC profile and GC-MS metabolite pattern in order to identify the bioavailable metabolites present in the extract.

## Materials and Methods

### Plant Materials and Extract Preparations

The leaves of *M. oleifera* were obtained from local market of Delhi and authentication was done as per protocol mentioned in Ayurved Pharmacopoeia. A voucher specimen with the number BNPL/JH/WK/02-04/2015 was deposited in our laboratory for future reference. The plant material was freed from foreign matter and grounded into coarse powder. The powder (100 g) was extracted through maceration with continuous stirring for 24 h using distilled water. The extract was filtered and subjected to lyophilize to get the solid residue. The obtained dried aqueous extract (AEMOL) was stored at 4°C until its use.

### Estimation of Total Phenolic and Flavonoid Content

The total phenolic content in the AEMOL was quantified by the Folin–Ciocalteu method (Kaur and Kapoor, 2002). Different concentration of standard (gallic acid) in place of the sample was used to prepare calibration curve which was further used for the calculation of total phenolic content. The results of total phenolic content of AEMOL was expressed as gallic acid equivalent (milligram of gallic acid per gram dry weight extract).

The total flavonoid content of the AEMOL was determined by the aluminum chloride colorimetric method (Saeed et al., 2012). The total flavonoid content in AEMOL was calculated from a calibration curve of standard (rutin), and the result was expressed as mg rutin equivalent to per gram dry weight.

### Quality Control Analysis of AEMOL through HPLC Fingerprinting

Twenty-five milligrams of AEMOL was dissolved in 5.0 mL of volumetric flasks using HPLC grade methanol to get 5.0 mg/mL solutions. Prepared solutions were filtered and 10 μL of solutions was injected for HPLC analysis. The HPLC analysis was carried out with the use of Alliance HPLC system (e2695 Separation module, Waters, United States) equipped autosampler attached with a PDA detector (Waters 2998), a gradient pump and the Empower software for data acquisition and processing. The analyses were carried out for 10 μL of AEMOL solution in methanol in the gradient mode using a Hypersil C18 (5 μ particle size) column (150 mm × 4.6 mm; Phenomenex, United States). Acetonitrile (A) and water with 0.5% (v/v) formic acid (B) were used in gradient elution program: from 0 to 7 min, 20% A; from 8 to 13 min, 40% A; from 14 to 19 min, 60% A; from 20 to 36 min, 100% A; and from 37 to 40 min, 20% A. The mobile phase flow rate was set to 1.0 mL/min and total run time of 45 min. The chromatograms were recorded with the use of PDA detector in scanning mode using the 3D channel at specific wavelengths were set at 254, 366, and 540 nm. The total number of peaks were identified for the quality aspects of the extract. Gallic acid, quercetin, and ellagic acid were identified by matching retention time of these standards in the extract chromatogram.

### *In Vitro* α-Amylase and α-Glucosidase Inhibition Assay

α-Amylase (A3176, Sigma) activity was carried out as per the method (Chukwuma and Islam, 2015), with minor modifications. Briefly, 1.0 mL of sample (AEMOL or acarbose) and 1.0 mL α-amylase (4 units/mL in sodium phosphate buffer, pH 6.7) were mixed and incubated for 30 min at 37°C. Further, 1.0 mL starch (1% w/v in the buffer) was added to the reaction mixture, and the whole mixture was then incubated for 1 h at 37°C. Further, 100μL of supernatant was taken out and glucose concentration was measured by glucose reagent (Erba Glucose, Erba, Germany). The α-amylase activity of AEMOL was determined by estimating the glucose concentration as per the protocol. Different concentrations of extract (10–500 μg/mL) were prepared in dimethyl sulfoxide and tested for α-amylase inhibition potential.

α-Glucosidase (G5003, Sigma) inhibition activity was carried out according to the previously reported method (Chukwuma and Islam, 2015), with slight modifications. Briefly, 120 μL sample (AEMOL or acarbose) and 20 μL of 1 unit/mL α-glucosidase in 0.1 M potassium phosphate buffer (pH 6.8) were incubated for 15 min at 37°C. The reaction was initiated by adding 20 μL of 5 mM *para*-nitrophenyl-α-D-glucopyranoside prepared in 0.1 M potassium phosphate buffer and the mixture was further incubated for 15 min. The reaction was terminated by adding 80 μL of 0.2 M Na_2_CO_3_ in 0.1 M potassium phosphate buffer, and then absorbance was measured at 405 nm.

The results were articulated as % inhibition of enzyme activity and calculated by the underneath equation:

%Inhibition = (absorbance of control−absorbance of sample)×100/absorbance of control

### *In Vitro* Antioxidant Activity

The antioxidant activity of the AEMOL was determined by 1,1-diphenyl-2-picrylhydrazyl (DPPH) assay, as described earlier (Vongsak et al., 2013) with some modifications. Briefly, 200 μL of extract (100–500 μg/mL) were mixed with 3.8 mL of DPPH solution and incubated in the dark at room temperature for 1 h. The absorbance of the solution was then recorded at 517 nm. Ascorbic acid was used as a positive control. The ability of the sample to scavenge the DPPH radical was calculated as indicated by the underneath:

DPPH scavenging effect = (control OD−sample OD)×100/control OD

### *In Vitro* Glucose Uptake Assay in Yeast Cell

The commercial baker’s yeast, *Saccharomyces cerevisiae* was grown in YM medium (1% dextrose, 0.5% peptone, 0.3% malt extract, 0.3% yeast extract, 2% agar) and the culture was centrifuged at 3,000 × *g* for 5 min. The supernatant was discarded and yeast cell pellet was taken for the experiment. Further, a 10% (v/v) of yeast suspension was prepared in sterilized deionized water. Various concentrations of AEMOL (50–500 μg/mL) were added to 1.0 mL of glucose solution (5, 10, and 25 mM), separately and incubated together at 37°C for 10 min. Glucose uptake was initiated by adding 100 μL of yeast suspension in each extract-glucose solution. The mixture suspension was vortexed and further incubated at 37°C for 60 min. After 60 min of incubation, the suspension was centrifuged (3,000 × *g*, for 5 min) and the supernatant was used to estimate the amount of glucose present (Nair et al., 2013). In yeast kinetic assay, *V*_max_ and *K*_m_ were calculated for both controls as well as the treatment group. Detailed procedure for the yeast cell kinetic assay has been described in “kinetic study of *ex vivo* glucose uptake.” The percentage increase of glucose uptake in yeast cells by using AEMOL was calculated using the underneath formula:

Increase in uptake(%) = (absorbance of sample−absorbance of control)×100/absorbance of sample

Where, absorbance of control reaction containing all reagents excluding the test sample; absorption of sample reaction containing AEMOL.

### *Ex Vivo* Glucose Uptake

#### Inhibition of Intestinal Glucose Uptake Assay

Five adult male Wistar rats with average body weight 250 ± 11.25 g were obtained from Central Animal Facility of Jamia Hamdard. The animals were fasted overnight (12 h) and euthanized by halothane anesthesia. The diaphragm was detached and dipped immediately in ice-cold Krebs–Henseleit buffer (NaHCO_3_, 25 mM/L; NaCl, 118 mM/L; KCl, 4.7 mM/L; MgSO_4_, 1.2 mM/L; CaCl_2_, 1.2 mM/L; Na_2_EDTA, 9.7 mM/L), previously equilibrated with 95% oxygen, 5% carbon dioxide. Each hemidiaphragm was cut transversely, yielding two pieces in all. Each piece of hemidiaphragm was blotted and transferred to the small cylindrical vessels containing a chilled medium. Different concentration of AEMOL (25–100 μg/mL) was also added in the same compartment separately. At the end of incubation (1 h, at 37°C), the sample was collected and concentration of unabsorbed glucose was estimated using a commercially available glucose estimation kit (Erba Glucose, Erba, Germany).

#### Effects of Glucose Uptake in Isolated Rat Skeletal Muscles

Dissected animals were used for the collection of parts of the skeletal muscle. The abdominal wall was dissected and the whole gastrointestinal tract was removed. Parts of the skeletal muscles was collected and immediately used for glucose uptake assay. The effect of AEMOL on glucose uptake in isolated rat skeletal muscles was determined according to the previously described method (Chukwuma and Islam, 2015) with minor modifications. Briefly, the collected skeletal muscle was instantly rinsed with Krebs buffer and cut into small pieces of equal weight (2.0 g). Each piece of skeletal muscle was then incubated in 10 mL of Krebs buffer, containing 20 mM glucose (control) and increasing concentrations of AEMOL (25–100 μg/mL) as a test or 100 μg/mL metformin solution in water was used as a positive control. The incubation period was for 1 h in an organ bath with a constant supply of 5% CO_2_, 95% O_2_ while maintaining the experimental temperature at 37°C. Before and after the incubation, 1 mL aliquot was collected from each experiment and the unabsorbed glucose concentration was measured. Glucose uptake in muscle was calculated as the amount of glucose (milligram) uptake by per gram of muscle tissue using the following formula:

Muscle glucose uptake = (GC1−GC2)/2.0⁢ g of muscle tissue

Where GC1 and GC2 are the concentration of glucose before and after incubation, respectively.

#### Kinetic Study of *Ex Vivo* Glucose Uptake

The kinetic studies were conducted to understand the transport in skeletal muscle and inhibition of glucose across intestinal membrane. In enzyme kinetics, the velocity of a reaction or transfer was an analog to the amount of glucose transported, which was measured in terms of concentration difference of the glucose at the beginning and end of an experiment (Therasa et al., 2014). The Michaelis–Menten constant (*K*_m_) and the maximal velocity (*V*_max_) were estimated from the differences in the glucose uptake and release values using Michaelis–Menten and the Lineweaver–Burk plots in Microsoft excel. Glucose as a substrate with different concentration ranging from 1 to 10 mg/mL was used both control and AEMOL-treated groups. The concentration of glucose in experimental solution was estimated at the end of incubation (for 1 h at 37°C). The difference in kinetic parameters between the control and test groups were compared and analyzed using one-way analysis of variance (ANOVA). In each run of experiments, intestinal membrane and skeletal muscle obtained from the same rat in a buffer without substrate (glucose) were performed in parallel. Controls were run without AEMOL and results obtained were corrected consequently.

### Animals, Diets, and Experimental Design

Female Wistar rats weighing about 200–250 g and mice (C57BL/6) of 20–25 g were used in our study. Both rats and mice were accustomed to laboratory conditions for 2 weeks and housed in polypropylene cages under standard laboratory conditions (light period: 12:12 h light and dark cycle; temperature: 23 ± 2°C; and relative humidity 55 ± 5%). The animals were fed a commercial diet (Hindustan Lever Ltd., Bangalore, India) and had provided free access to water (*ad libitum*) during the experiments. The experiments were conducted under strict guidelines of the Committee for the Purpose of Control and Supervision of Experiments on Animals, New Delhi, India (Registration No.: 173/GO/RE/S/2000/CPCSEA) and the study was permitted (Approval No. 1206) by the Institutional Animal Ethical Committee of the Hamdard University, New Delhi, India.

### Streptozotocin-Induced Diabetes

A single dose of 45 mg/kg body weight of freshly prepared streptozotocin (STZ, S0130, Sigma-Aldrich) in 0.1 mol/L citrate buffer (pH 4.5) through intraperitoneal route was used for the induction of diabetes in overnight fasted rats. After 72 h of STZ treatment, diabetes mellitus was developed in the test groups, which was confirmed by assessing fasting blood glucose (FBG) from the tail vein of overnight fasted rats. Rats exhibiting blood glucose of 250 mg/dL or higher were reflected to be diabetic and were encompassed in our study (Kaur et al., 2016). This day was considered as the 1st day of our experiment. Further, rats were divided into five groups (*n* = 6 per group). Group 1 received normal saline (NS) and served as control; Group 2 received AEMOL (100 mg/kg) for 3 weeks and served as sham control whereas Groups 3–5 treated with STZ to induce diabetes. In addition to STZ treatment, Group 3 received NS and served as toxic control; Group 4 received AEMOL (100 mg/kg) and served as a test; Group 5 received metformin (42 mg/kg) and served as positive control. The dose of the AEMOL was calculated from the extractive value obtained with respect to the dose mentioned in Ayurved Pharmacopoeia. Both acute and chronic effect of the extract on blood glucose in normal and diabetic rats were assessed. The acute hypoglycemic effect of the AEMOL was measured by assessing blood glucose level at different time intervals, i.e., 0, 1, 2, 3, 4, 6, 8, 10, and 12 h after drug administration for two consecutive days whereas, in chronic effect, FBG was measured on each day up to 3 weeks after 4 h of drug administration. At the end of the experiment, rats were sacrificed using ether anesthesia. Before scarification of rat, blood samples were collected from a retro-orbital vein for further biochemical analysis.

### High-Fat Diet Induced Diabetes

Adult female C57BL/6 mice of 6 weeks age were used for this study. High-fat diet (HFD) was freshly prepared by lard obtained from sheep and normal commercial pellet diet, which was further used for the induction of diabetes upon administration for a period of 8 weeks (Huang et al., 2016). Development of diabetes was confirmed by polyuria, increase in weight and blood glucose level in between the study duration, randomly. Mice with blood glucose level of 150 mg/dL or higher were considered as diabetic and selected for the experiment. Blood glucose level was measured using blood glucose monitoring system (On Call Plus Blood Glucose Meter, ACON Laboratories, United States) from the tail vein. A total of 30 mice were used in this study, out of which 18 mice were diabetic and 12 were normal. Mice were divided into five groups and six mice in each group. Group 1 (control) received standard diet only; Group 2 (Sham control) received 200 mg/kg AEMOL daily for 3 weeks with the standard diet. Groups 3–5 were HFD-induced diabetic mice. Group 3 (toxic) received NS only; Group 4 (test) received 200 mg/kg AEMOL daily for 3 weeks; Group 5 (positive control) received 85 mg/kg metformin. The dose of the AEMOL was calculated from an extractive value obtained with respect to the oral dose mentioned in Ayurved Pharmacopoeia. The level of blood glucose was checked daily during the period of the study and 7 days after the completion of the study.

### Oral Glucose Tolerance Tests in Rats

Oral glucose tolerance test (OGTT) was carried out in the overnight fasted diabetic rat. Animals were divided into two major categories on the basis of blood glucose level, i.e., mild-diabetic and sub-diabetic. The animals were treated with AEMOL (100 mg/kg, p.o.) as test and metformin (42 mg/kg, p.o.) as standard. However, control group administered the vehicle only. FBG was checked initially, and then blood glucose was taken after 30 min of treatment with extract considered as “0 h” value. Further, rats were orally administered with glucose (2.0 g/kg) and their glucose tolerance as blood glucose level was studied up to 6 h at regular intervals (0, 0.5, 1, 2, 3, 4, 5, and 6 h). The area under the curve (AUC) were calculated using the trapezoidal method to check the glucose responses during the OGTT with the help of Excel software plug-in program pkf (Joel I. Usansky, Atul Desai, and Diane Tang-Liu, Department of Pharmacokinetics and Drug Metabolism, Allergan, Irvine, CA, United States).

### Evaluation of Biochemical Parameters

At the end of the experimental period, the overnight fasted animals (both rats and mice) were sacrificed using ether anesthesia. Blood samples were collected from the retro-orbital vein and the serum was obtained by centrifugation (2,000 × *g* for 20 min) and stored at -20°C for further assay. The activities of aspartate aminotransferase (SGOT), alanine aminotransferase (SGPT), and lipids levels [total cholesterol (TC), triglycerides (TG), high-density lipoprotein (HDL), low density lipoprotein (LDL), and very low density lipoprotein (VLDL)] in the serum were analyzed by using commercial diagnostic kits (for SGOT and SGPT—Span diagnostic Ltd., Gujarat, India; for lipid profiles—Erba reagent for lipid profile, Erba, Germany).

### Pattern Recognition of Metabolites of AEMOL through GC-MS Metabolomics

Healthy Wistar rats were used for *in vivo* pattern recognition of metabolites present in the AEMOL. Extract at a dose of 100 mg/kg was orally administered and blood samples were collected at different time intervals (0, 1, 2, 4, 8, and 12 h) from a retro-orbital vein. Collected blood samples were subjected to centrifuge at 4,000 rpm for 10 min and serum was separated (Khan et al., 2016a,b).

Prior to GC-MS analysis, the serum samples of different time intervals were extracted to separate polar and lipophilic metabolites separately. For, extraction of polar metabolites, briefly about 100 μL of serum aliquot was extracted with 800 μL of HPLC grade methanol: HPLC grade water (8:1, v/v) and vortexed for 2 min. The mixture was kept at 4°C for 25 min and then centrifuged at 4,000 rpm for 10 min. After centrifugation, the supernatant was evaporated to dryness using N_2_. For extraction of lipophilic metabolites, briefly about 100 μL serum sample was mixed with 750 μL chloroform, 200 μL methanol, and 100 μL of 0.5 M KH_2_PO_4_ and vortexed for 2 min. The resulting solution was kept at 4°C for 25 min and then centrifuged at 4,000 rpm for 10 min. The supernatant was removed and the chloroform layer was collected. The chloroform layer was washed with water and evaporated to dryness under N_2_. Metabolites of AEMOL were suspended in methanol and then the methanolic solution was extracted with HPLC grade chloroform to get lipophilic metabolites of AEMOL. However, AEMOL was used as such for analysis of polar metabolites.

Both polar and lipophilic metabolites extracted from serum and from AEMOL were reconstituted in 100 μL of 20 mg/mL methoxamine hydrochloride (SLBP2736V, Sigma-Aldrich) in pyridine (STBF5486V, Sigma-Aldrich) separately. Further, it was kept at room temperature for 16 h, then 100 μL of *N*-methyl-*N*-trifluoroacetamide (BCBL0296, Sigma-Aldrich) with 1% trimethylchlorosilane as a derivatization reagent was added and incubated at 37°C for another 2 h and intermittently vortexed with a regular interval to ensure complete dissolution. Derivatized samples were analyzed within 2 days (Psychogios et al., 2011).

Derivatized samples were analyzed by GC-MS instrument (Agilent 7890A, Agilent Technologies, United States) equipped CTC-PAL auto sampler attached with a Mass spectrophotometer detector (Agilent 5975C inert XL EI/CI MSD with Triple-Axis Detector, Agilent Technologies, United States). A 2.0 μL of derivatized sample was injected with a 10:1 split ratio onto a 30 m × 0.25 mm × 0.25 μm HP-5_MS_ column (5% diphenyl, 95% dimethyl polysiloxane, Agilent Technologies, United States). The oven temperature program was set as follows: 50°C initially for 1 min, increased to 150°C at 5°C/min and the temperature was held for 1 min. Then it was ramped to 310°C at 10°C/min; 310°C was maintained for 5 min. Total run time of sample run was 43 min. High pure helium (99.999%) was the carrier gas set at a constant flow rate of 1 mL/min. The injection port, transfer line, and ion source temperatures were all set at 250°C and the helium gas flow rate was set to 1 mL/min at an initial oven temperature was held at 50°C. 70 eV of EI will be adopted, and the mass scanning range was set from 50 to 700 amu in full scan. Solvent delay time was set for 3 min for all samples generated by different methods. MSD ChemStation software will be used to process data. The metabolite separated through GC-MS was identified by comparing the obtained mass spectra of the analytes with those of authentic standards from the NIST libraries (2005) and with the mass spectra published previously. Peak areas of all components were calculated by MSD ChemStation, and relative amounts of volatile compounds was calculated on the basis of peak-area ratios (Psychogios et al., 2011).

### Statistical Analysis

All the experiments were performed in triplicate and data were indicated as mean ± SEM. Results were analyzed by two-way ANOVA, and the level of significant differences among different groups was determined by Bonferroni post-tests using GraphPad Prism, ver 5.00 software. At 95% confidence interval, *p* < 0.05 considered as statistically significant.

Principal component analysis (PCA) is a powerful statistical tool used for analysis, pattern identification, and expression of data, which can enable the emphasizing of a group’s similarities and differences (Berrueta et al., 2007). It can be presumed that the content and diversity of metabolites in AEMOL and in blood at different intervals should be different. Not all the metabolites are going to be absorbed after oral administration and it might possible that metabolites are being transformed in other form and further it was bioavailable. Therefore, it can be presumed that metabolites of aqueous extract and in blood at different time intervals were considered as having different sets of metabolites. For PCA, the chromatograms of GC-MS analysis were transformed into a table (a matrix) of peak intensities of metabolites. In this table (**Supplementary Tables S1**, **S2**), a row corresponds to certain metabolites identified by comparing spectrum from NIST library and a column represents sources of metabolite obtained.

Comparative metabolite analysis of metabolites obtained from the GC-MS analysis was carried out to find the similarity in metabolic profiling in blood after oral administration of AEMOL. Data was normalized as per the presence (value 1) or absence (value 0) of metabolite analyzed at different time intervals. The normalized data was further analyzed by the XLSTAT 2014.4.08 PCA demo version for multivariate data analysis and for similarity analysis. The Extended Statistics (XS) module of the XLSTAT software was used to perform multivariate statistical analysis of the both polar and lipophilic metabolite dataset and using this module, a heatmap analysis was conducted to visualize the relationships and relative levels of metabolites present in AEMOL and in blood samples collected at different time intervals. Heatmap was created using all 62 polar and 84 lipophilic metabolites analyzed from AEMOL and in blood samples.

## Results

**FIGURE 1:**
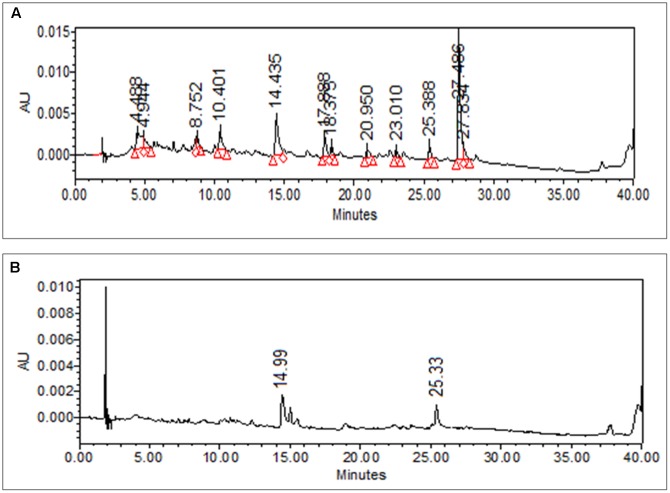
HPLC chromatogram of methanolic solution AEMOL at **(A)** 254 nm and **(B)** 360 nm.

The dried leaves of *M. oleifera* were extracted using distilled water by maceration and extract obtained called mother extract having yield value of 38.5% w/w. The extract obtained was slightly gummy in nature and black in color. **Figure 1** shows the HPLC chromatogram of AEMOL recorded at two different wavelengths (254 and 340 nm). At 254 nm, 11 major peaks were observed at R_t_ 4.48, 8.75 (gallic acid), 10.4, 14.43 (rutin), 17.88, 18.37, 20.95, 23.01, 25.38, 27.48, and 27.83 min (**Figure 1A**), whereas at 340 nm only two peaks have been identified, which were eluted at 14.99 and 25.38 min (**Figure 1B**). At a wavelength of 540 nm, no peak has been detected.

### Total Phenolic and Flavonoid Content

The total phenolic content of the AEMOL was calculated from the calibration curve (*R*^2^ = 0.998) and it was 31.62 ± 3.19 g of gallic acid equivalents per 100 g of extract. Similarly, a calibration curve of rutin (*R*^2^ = 0.999) was used to express total flavonoid content and it was 27.32 ± 1.76 g of rutin equivalents per 100 g of extract. Phenolic compounds have redox properties for which it acts as antioxidants (Soobrattee et al., 2005). The total phenolic content could be used as a basis for rapid screening of antioxidant activity because of their hydroxyl group which facilitated the free radical scavenging ability. The antioxidant activity of flavonoids which include flavones, flavanols, and condensed tannins depends on the presence of free OH groups, especially 3-OH. Plant flavonoids and phenolics are responsible for both *in vitro* and *in vivo* antioxidant activity (Shimoi et al., 1996; Geetha et al., 2003). Since the present report of antioxidant activity of AEMOL suggesting that a complete phytochemical profiling needs to be done to identify the other active phenolic and flavonoid components.

### *In Vitro* α-Amylase and α-Glucosidase Inhibition Activity

**FIGURE 2:**
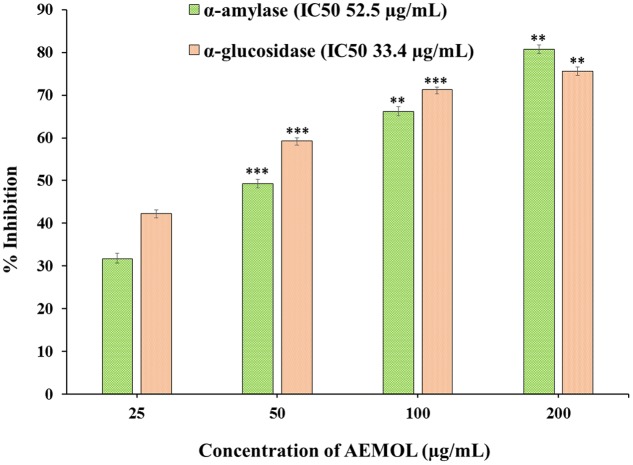
Graphical representation of α-amylase and α-glucosidase inhibition potential of AEMOL. Data were represented as mean ± SD; ^∗∗^*p* < 0.01, ^∗∗∗^*p* < 0.001.

AEMOL has excellent α-amylase and α-glucosidase inhibition potential. The inhibitory effect of AEMOL against α-amylase and α-glucosidase were followed in a dose-dependent manner, and the average inhibitions were 56.96 and 62.1%, respectively (**Figure 2**). At low concentration, α-glucosidase inhibition followed in a dose-dependent manner but at higher concentration enzyme inhibition activity reached to a constant level. Maximum α-amylase (80.5%) and α-glucosidase (75.65%) inhibition was found at 200 μg/mL of extract solution. The IC_50_ values of the AEMOL against α-amylase and α-glucosidase were 52.5 and 33.4 μg/mL, respectively. Lower IC_50_ values indicating the better potentiality of AEMOL.

### Antioxidant Potential of AEMOL

AEMOL are rich in secondary metabolites, including phenolics and flavonoids which have antioxidant activity due to their redox properties. The AEMOL had shown strong antioxidant potential against all the free radicals. Due to the ease of reaction, DPPH radical is commonly used to determine the free radical scavenging activity of any plant extract. Maximum DPPH scavenging activity was 55.1% observed at a concentration of 100 μg/mL of AEMOL, while that of the control, ascorbic acid, was 83.42%. The DPPH scavenging potential of AEMOL was increased with respect to the concentration of extract up to 100 μg/mL. Further, the increment of extract concentration (up to 125 μg/mL) resulting in no increment of DPPH scavenging potential. Flavonoids are highly powerful scavengers of most oxidizing molecules which include singlet oxygen and various other free radicals associated with several diseases. Flavonoids suppress the reactive oxygen formation, scavenge reactive species, up-regulate antioxidant defenses and chelated trace elements which involved in the free-radical production (Agati et al., 2012). Similarly, phenolic metabolites conferred the oxidative stress tolerance in plants (Shukla et al., 2009).

### *In Vitro* Glucose Uptake Assay in Yeast Cell

**FIGURE 3:**
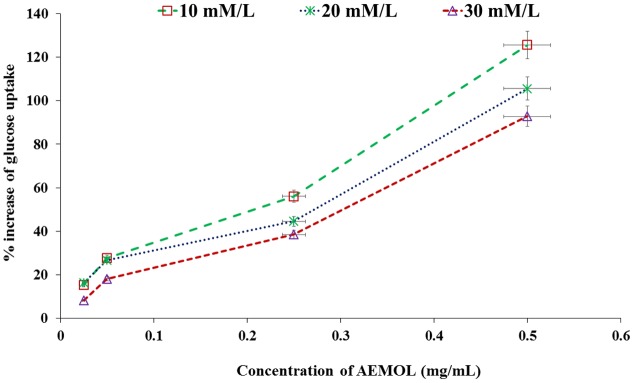
Effect of AEMOL extract on the uptake of glucose by yeast cells. All values were represented as mean ± SD of triplicate determinations.

Through facilitated diffusion, glucose transport takes places in baker’s yeast. The amount of glucose remains in the experimental medium after a specific incubation time period indicates the glucose uptake by the yeast cell. Glucose uptake in yeast cell was increased in a dose-dependent manner when yeast cell was supplemented with AEMOL. **Figure 3** depicts the percent increase in glucose uptake by the yeast cells at different glucose concentrations, i.e., 10, 20, and 30 mM with respect to extract concentration. Results also indicated that AEMOL had greater efficiency in increasing the glucose uptake by yeast cells as compared to positive control. However, the percent increase of glucose uptake in the yeast cells by extract was observed as directly proportional to the extract concentration while it remained constant when molar glucose concentration was increased. A significant difference (*p* < 0.001) has been observed in glucose uptake in comparison to the positive control. An increase in *V*_max_ and *K*_m_ was also observed in AEMOL-treated experimental group (*V*_max_ 0.55 and *K*_m_ 10.33) as compared to control group (*V*_max_ 0.38 and *K*_m_ 13.95) indicating rate increase in glucose uptake in yeast cell incubated with a solution of AEMOL which also supports the results of yeast cell uptake assay.

### Effects of AEMOL on Intestinal Glucose Absorption Inhibition Assay

**FIGURE 4:**
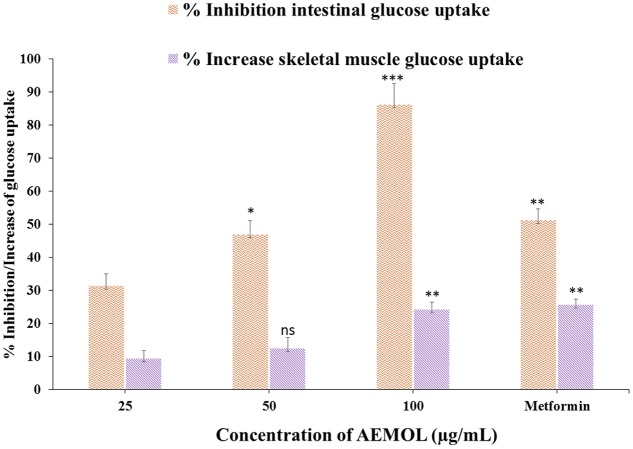
Effect of AEMOL on *ex vivo* parameters of the transport of glucose at different concentrations (5.5–8.5 mg/dL) across the rat intestinal membrane and skeletal muscle. Data were represented as mean ± SD; ns *p* > 0.05, ^∗^*p* < 0.05, ^∗∗^*p* < 0.01, ^∗∗∗^*p* < 0.001.

Many investigators have used the isolated diaphragm to check the effect of insulin-mimetic substances on insulin based stimulation of glucose uptake in muscle tissue. In the present study, the effect of the AEMOL on intestinal glucose uptake has been investigated. A marked decrease in glucose uptake through the intestinal membrane by AEMOL has been observed as compared to control. Inhibition of glucose uptake follows in a dose-dependent manner (**Figure 4**). Maximum (86.25%) glucose uptake inhibition has been observed at a concentration of 100 μg/mL of AEMOL. The kinetic study resulted in a significant (*p* < 0.05) decrease in *V*_max_ in the case of AEMOL-treated experiment (*V*_max_ 0.37 and *K*_m_ 3.09) as compared to control group (*V*_max_ 0.74 and *K*_m_ 3.78) but no significant difference has been observed in *K*_m_.

### Effects of AEMOL on Glucose Uptake by Isolated Rat Skeletal Muscle

The effects of AEMOL on glucose uptake in isolated rat skeletal muscle is shown in **Figure 4**. Increase in glucose uptake by skeletal muscle was observed with increasing concentrations of the AEMOL and a maximum 24.3% glucose uptake was increased at a dose of 100 μg/mL of extract as compared to control. The effect of AEMOL and standard was similar and a significant change (*p* < 0.05) in glucose uptake was observed as compared to the control. The kinetic study revealed that increment in *V*_max_ in the case of AEMOL-treated experiment (*V*_max_ 0.31 and *K*_m_ 1.69) as compared to control group (*V*_max_ 0.29 and *K*_m_ 1.74) but no significant difference has been observed in *K*_m_. Increased in glucose uptake in muscle might be due to the enrichment of phenolic content. Previously, it has been reported that polyphenol-rich extract activates both phosphatidylinositol-3 kinase (PI3K) and 5′ adenosine monophosphate (AMP)-activated protein kinase (AMPK) signaling in muscle cells. The results showed that AEMOL has better glucose uptake potential than metformin and it may be because of activation of AMPK (Zou et al., 2004). Thus, the present model can be used for the discovery of novel bioactive phytopharmaceutical that stimulates glucose uptake in muscle. Hence, it could provide alternative approaches for the treatment of insulin resistance and type 2 diabetes.

### Kinetic Study of *Ex Vivo* Glucose Uptake

The value of *V*_max_ was significantly decreased in case of intestinal glucose absorption and increased in glucose uptake in skeletal muscle when incubated with the AEMOL as compared with the control but no significant changes have been observed in *K*_m_ (**Table 1**). The decreased in the *V*_max_ in the presence of the AEMOL indicating a significant decrease in the transmembranal glucose transport intestinal membrane. However, the *K*_m_ remained unchanged throughout the study. This indicates that the AEMOL acts as a non-competitive inhibitor of transport of glucose at the level of the intestine and in skeletal muscle which may be because of inhibition of glucose transporter proteins (GLUT2 and GLUT4) activity.

**Table 1:** Effect of AEMOL on kinetic parameters of the transport of D-glucose at different concentrations (5.5–8.5 mM/L) across the rat intestinal membrane and skeletal muscle.

	Intestinal glucose		Skeletal muscle	
	uptake inhibition		uptake	
Experiments	*V*_max_ (mM/h)	*K*_m_ (mM)	*V*_max_ (Mm/h)	*K*_m_ (mM)
Control (*n* = 6)	0.74 ± 0.02	3.78 ± 0.04	0.29 ± 0.01	1.74 ± 0.03
AEMOL (*n* = 6)	0.37 ± 0.01	3.09 ± 0.02	0.31 ± 0.01	1.69 ± 0.02

**K*_*m*_ and *V*_*max*_ values were obtained from Lineweaver–Burk plot. *K*_*m*_, Michaelis–Menten constant, which is the affinity of the transferring enzyme for the substrate. *V*_*max*_, maximal velocity, which is the rate of transfer reaction, in the presence as well as in the absence of AEMOL determined from the differences of uptake and release values using Michaelis–Menten and Lineweaver–Burk plots in Microsoft Excel*.

### Effect of AEMOL on STZ-Induced Hyperglycemia

**Table 2** describes the acute hypoglycemic effect of AEMOL on fasting glucose of STZ-induced and normal rats. Rats treated with 100 mg/kg of AEMOL, showed a maximum fall of 53.2% in fasting glucose after 4 h of oral administration whereas the fall of 40.1, 45.6, and 41.7% was observed after 3, 6, and 8 h, respectively on day 1 and day 2. In diabetic rats, as shown in **Table 2**, both AEMOL and metformin had a time-dependent hypoglycemic activity. But maximum levels of blood glucose brought down by 42.4% at 6th hour for metformin as compared with the diabetic control group.

**Table 2:** Acute effect of AEMOL on STZ-induced diabetes.

	Control		Toxic		AEMOL		Metformin		Sham	
H/day	Day 1	Day 2	Day 1	Day 2	Day 1	Day 2	Day 1	Day 2	Day 1	Day 2
0	78.8 ± 3.0	79.1 ± 3.6	265.0 ± 5.4^∗∗∗^	266.0 ± 4.9^∗∗∗^	224.5 ± 3.9^##^	233.3 ± 3.3^##^	264.5 ± 1.7^ns^	264.8 ± 1.8^ns^	81.2 ± 3.1^ns^	79.3 ± 2.5^ns^
1	78.5 ± 3.8	77.5 ± 5.4	270.0 ± 2.6^∗∗∗^	272.5 ± 2.1^∗∗∗^	201.6 ± 10.4^###^	188.5 ± 2.1^##^	266.1 ± 1.0^ns^	268.1 ± 2.1^ns^	82.3 ± 2.4^ns^	81.3 ± 5.4^ns^
2	78.8 ± 4.7	80.0 ± 5.4	273.6 ± 2.2^∗∗∗^	274.5 ± 2.6^∗∗∗^	175.0 ± 9.4^###^	151.3 ± 4.4^##^	208.5 ± 3.1^###^	212.0 ± 4.0^###^	80.5 ± 1.5^ns^	80.3 ± 27^ns^
3	80.1 ± 3.1	81.1 ± 4.0	271.5 ± 4.2^∗∗∗^	271.3 ± 3.8^∗∗∗^	160.5 ± 7.5^###^	141.0 ± 6.5^###^	197.6 ± 0.8^###^	198.3 ± 1.5^###^	81.2 ± 2.6^ns^	79.5 ± 5.4^ns^
4	79.3 ± 4.1	81.0 ± 2.9	273.8 ± 2.8^∗∗∗^	276.0 ± 2.1^∗∗∗^	128.5 ± 5.6^###^	118.2 ± 3.1^###^	173.3 ± 1.3^###^	176.3 ± 1.2^###^	83.3 ± 3.1^ns^	81.6 ± 3.2^ns^
6	79.1 ± 5.3	78.5 ± 3.2	268.3 ± 2.3^∗∗∗^	271.1 ± 1.7^∗∗∗^	143.6 ± 8.8^###^	143.5 ± 3.1^###^	152.1 ± 3.3^###^	152.6 ± 5.7^###^	82.4 ± 2.5^ns^	81.6 ± 4.5^ns^
8	78.5 ± 3.7	77.8 ± 3.6	271.5 ± 1.1^∗∗∗^	271.5 ± 1.1^∗∗∗^	158.2 ± 8.4^###^	167.6 ± 5.7^###^	214.6 ± 1.2^##^	216.1 ± 1.8^###^	83.5 ± 4.5^ns^	80.4 ± 3.6^ns^
10	77.1 ± 3.2	77.3 ± 2.9	272.6 ± 2.3^∗∗∗^	273.3 ± 2.6^∗∗∗^	244.0 ± 10.8^###^	241.6 ± 3.6^###^	223.5 ± 0.9^##^	225.8 ± 1.5^###^	79.7 ± 2.6^ns^	78.4 ± 3.6^ns^

*Data were expressed as mean ± SEM; ns *p* > 0.05, ^∗^*p* < 0.05, ^∗∗^*p* < 0.01, ^∗∗∗^*p* < 0.001; asterisk “^∗^” denoting that data were compared with normal control and hash “#” denoting that data were with toxic control. ^#^*P* < 0.05, ^##^*P* < 0.01, ^###^*P* < 0.001.*

**FIGURE 5:**
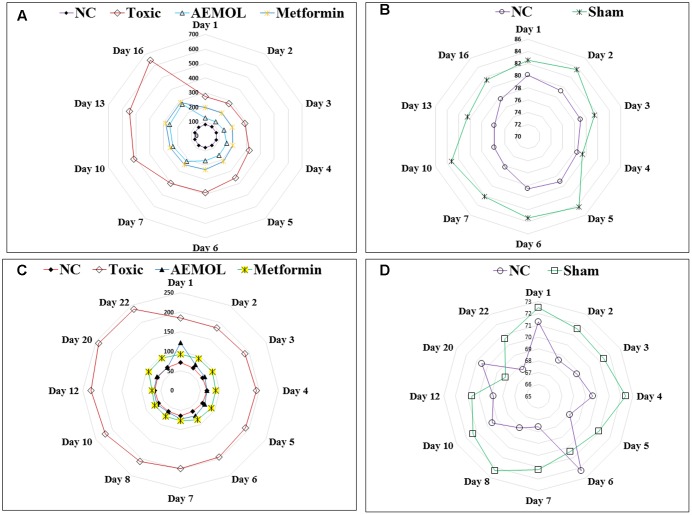
Radar graph showing the effect of AEMOL on blood glucose level in STZ-induced diabetic rats and HFD-induced diabetic mice; panel **(A)** showing variation of blood glucose level in toxic control, AEMOL-treated, metformin-treated, and normal control rats in STZ-induced diabetic rats, panel **(B)** showing the no significant differences between normal control and sham control rats, panel **(C)** showing variation of blood glucose level in toxic control, AEMOL-treated, metformin-treated, and normal control mice in HFD-induced diabetic mice, panel **(D)** showing the no significant differences between normal control and sham control mice.

The chronic effect of AEMOL on fasting glucose levels of STZ-induced diabetic rat has been shown in **Figures 5A,B**. Radar graph (**Figure 5A**) showing the differences in blood glucose observed in normal control and treated groups against STZ-induced diabetic rats. However, it has been clearly demonstrating that there are no significant differences in normal control and Sham control rats (**Figure 5B**), indicating no placebo effect of AEMOL on blood glucose level. It has been observed that control and sham control groups showed normal glucose level throughout the experiment and thus glucose level of the control group was used for comparative analysis with other groups. However, the mean fasting glucose of STZ group showed a higher blood glucose level, while in the AEMOL-treated group, the mean fasting glucose was significantly decreased (*p* < 0.05). From the 1st day, a significant fall in blood glucose level was observed as compared to toxic control. However, percentage fall of blood glucose level remained constant throughout the experimental period. Blood glucose level was increased daily but it was significantly lower as compared to the toxic control. One out of ten animals in STZ group died during the experimental period.

### Effect of AEMOL on HFD-Induced Hyperglycemia

The chronic effect of AEMOL on fasting glucose levels of HFD-induced diabetic mice has shown in **Figures 5C,D**. Radar graph (**Figure 5C**) showing the differences in blood glucose observed in normal control and other treatment groups of HFD-induced diabetes. It has been clearly demonstrated that there are no significant differences in Normal control and Sham control rats (**Figure 5D**), indicating no placebo effect of AEMOL on mice. The chronic effect of AEMOL on HFD-induced diabetic mice is shown in **Figures 5C,D**. After 2 days of treatment, the HFD-induced hyperglycemia was significantly (*p* < 0.01) ameliorated by the AEMOL. On 2nd day, blood glucose level was decreased about 34.23% and on 3rd day, it was decreased about 58.69% at a rate of 71.4% per day. It was observed that upon treatment with AEMOL, the blood glucose level of diabetic mice became normal on 2nd day and it was not further increased or decreased, while in HFD group it was continuously increasing. After a gap of 7 days of AEMOL treatment, the blood glucose level was still normal, concluding that this drug can cure completely HFD-induced diabetes.

### Oral Glucose Tolerance Test

**FIGURE 6:**
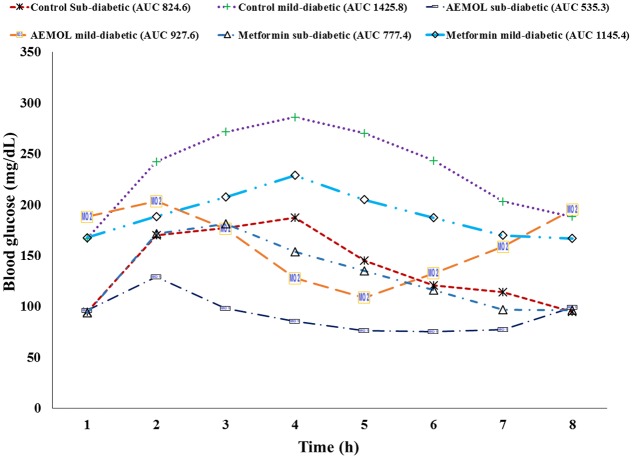
Effects of AEMOL on glucose tolerance test in mild and subdiabetic rats. Rats were administered with 2 g/kg glucose (*n* = 6) (control: saline). The extract was administered 30 min before glucose administration. Blood glucose was measured at the time of glucose administration and at 1, 1.5, 2, 3, 4, 5, and 6 h after glucose administration. All values were represented as mean ± SEM.

The change in blood glucose level in mild as well as in sub-diabetic rats after oral administration of glucose are shown in **Figure 6**. It demonstrates the effect of AEMOL on blood glucose of sub-diabetic and mild-diabetic rats during OGTT experiments. After 2 h of glucose administration, the maximum fall in blood glucose level observed with the dose of 100 mg/kg was 54.5 and 55.3% in case of sub-diabetic and mild-diabetic, respectively. Moreover, metformin, the reference drug produced a fall of only 45.18 and 24.11% in sub-diabetic and mild-diabetic cases, respectively and it was lesser than the fall produced by AEMOL. However, the blood glucose level was significantly (*p* < 0.001) decreased for the extract as well as metformin-treated in both diabetic and control group. Similarly, a significant (*p* < 0.001) difference has been observed in AUC of glucose responses during OGTT among all groups as compared with the control.

### Effect of AEMOL on Biochemical Parameters

**FIGURE 7:**
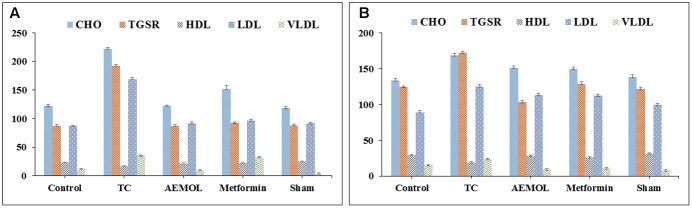
Changes in biochemical parameters of **(A)** HFD-induced mice and **(B)** STZ-induced rats.

The HFD group showed a significant (*p* < 0.05) increase in serum TC, TG, LDL, VLDL levels while a significant (*p* < 0.05) reduction in serum HDL level as compared to the control group (**Figure 7**) was observed. In this study, administration of AEMOL significantly reduced elevated TC, TG, VLDL, and LDL levels in HFD-induced diabetic mice. Also, an increase in the level of HDL was observed in diabetic mice treated with AEMOL and metformin treatment group as compared to diabetic control. But the level of restoration of lipid profile in STZ-induced rats was lower than HFD-induced mice. This result supports the lipid-lowering activity of AEMOL in diabetic condition and hence prevents diabetic-associated complications. No significant changes in biochemical parameters have been observed in normal control animals treated with AEMOL (sham control) when compared with normal control. The level of liver function marker enzyme (SGOT and SGPT) was significantly (*p* < 0.01) reversed in AEMOL-treated group as compared to both toxic groups (HFD/STZ-induced diabetic group).

### Pattern Recognition

**FIGURE 8:**
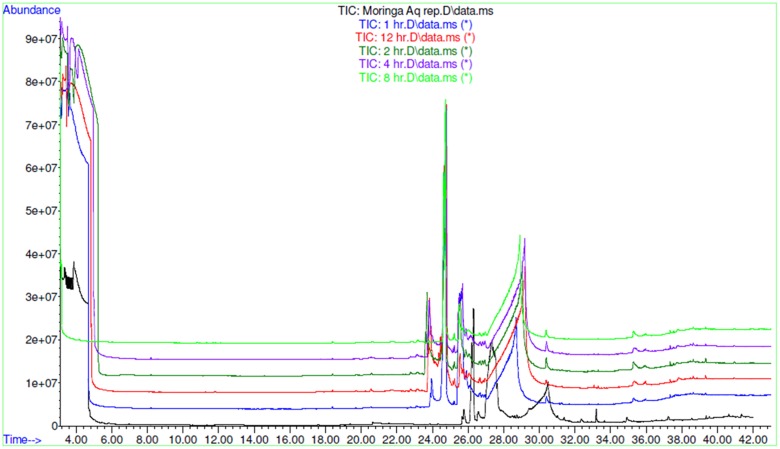
GC-MS overlay plot of polar metabolites in extract and in blood sample collected at different time intervals after oral administration of extract in rat at a dose of 100 mg/kg.

**FIGURE 9:**
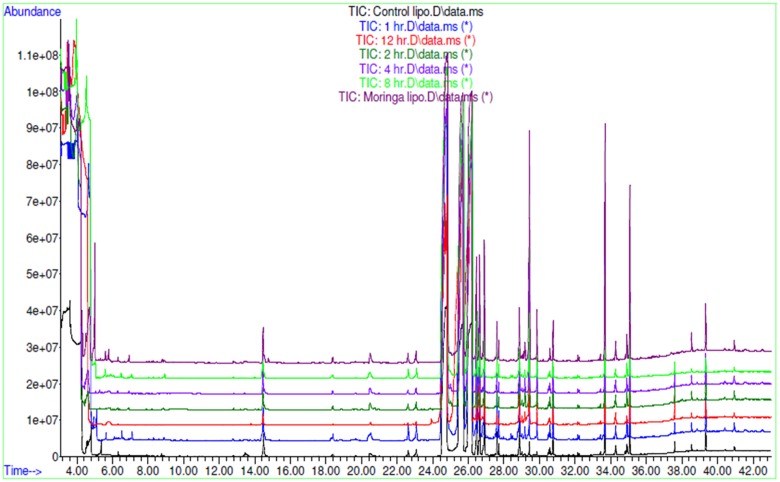
GC-MS overlay plot of lipophilic metabolites in aqueous extract and in blood sample collected at different time intervals after oral administration of aqueous extract in rat at a dose of 100 mg/kg.

AEMOL was orally administered to Wistar rat at a dose of 100 mg/kg and blood samples were collected at different time intervals. A total of 43 polar metabolites were identified in AEMOL, however, only nine metabolites were absorbed. The number of metabolites absorbed was varied with respect to the time intervals such as 4, 2, 1, 1, and 3 metabolites were absorbed in 1, 2, 4, 8, and 12 h of oral administration, respectively. Surprisingly, 19 new polar metabolites were found in blood which is not present in the AEMOL. In a similar way, lipophilic metabolites of AEMOL were also identified. Among 40 lipophilic metabolites present in the AEMOL, 25 were bioavailable and 45 new metabolites were found in blood. Thus, a total of 63 polar and 84 lipophilic metabolites were identified by GC-MS analysis of AEMOL and in blood. **Supplementary Tables S1**, **S2** showing the number of metabolites found in AEMOL and in a blood sample. Metabolites with their elution pattern, tentative name, and their abundance are shown as per GC-MS data. A GC-MS overlay plot of polar (**Figure 8**) and lipophilic (**Figure 9**) metabolites indicating the similarity and dissimilarity of metabolite pattern based on their elution pattern in AEMOL and in a blood sample. Metabolites were tentatively identified by matching their mass spectrum from NIST library. Characterization of metabolites pattern was evaluated based on their elution pattern.

## Discussion

In the traditional system of medicine, AEMOL has been used for various therapeutic purposes and recent scientific data are available for its others therapeutic properties such as antioxidant, anticancer, anti-inflammatory, antidiabetic, hypolipidemic, and hypotensive (Kajihara et al., 2008; Jaiswal et al., 2009; Amaglo et al., 2010; Jain et al., 2010; Minaiyan et al., 2014; Al-Asmari et al., 2015). As an antidiabetic agent, its leaves, pods, and seeds have been scientifically proven (Jaiswal et al., 2009; Gupta et al., 2012; Al-Malki and El Rabey, 2015). Most of the researchers have used alcoholic or hydroalcoholic extract of *M. oleifera* leaves but as per the recommendation of the traditional system of medicine, the only aqueous extract is preferable. In our present study, we demonstrated the AEMOL with a low dose for its antidiabetic potential. Thus our study will be preferable for the development of herbal therapy or novel phytopharmaceuticals from AEMOL which can be used for diabetes. By maceration with water yielding a higher percentage of extract and simple to extract which can be used in industrial scale also. In our study, we used a low dose of extract which was equivalent to dose mentioned in Ayurved Pharmacopoeia but we found a significantly higher antidiabetic potential than previously reported study (Jaiswal et al., 2009; Divi et al., 2012; Dawood and Fathy, 2014). AEMOL with a dose of 100 mg/kg reduces the blood glucose levels in the sub, mild, and severely diabetic rats. In hyperglycemic rat and mice, a maximum of 54.5% blood glucose was decreased by AEMOL, while previously it was 23.2% (Divi et al., 2012). However, in normoglycemic rat and mice, no significant changes in blood sugar level has been observed upon oral administration of AEMOL, concluding its safety which was proved by using a higher dose of AEMOL with no toxic or side effect (Awodele et al., 2012). STZ causes a pancreatic loss in rat while HFD caused increased insulin resistance and decreased in glucose sensitivity (King, 2012). Till date, no experimental model is available which can check its effect on pancreatic tissue, insulin resistance, and glucose sensitivity. Thus, both STZ and HFD model separately are still preferable for checking antidiabetic properties of the dual mode of antidiabetic drugs. In our study, we checked and compared the effect of AEMOL in STZ-induced diabetes in rats and HFD-induced diabetes in C57BL/6 mice. STZ-induced diabetes in rats caused enhancement of ATP dephosphorylation and with xanthine oxidase substrate resulting formation of superoxide radicals, hydroxyl radicals and hydrogen peroxide were formed (Al-Malki and El Rabey, 2015) and further, serious health problem illustrated as an increase in blood glucose. AEMOL ameliorated the blood glucose to normal level in acute as well as in chronic treatment and this effect may be due to the presence of phenolics and flavonoids which having the scavenging effect of free radicals produced by STZ, increasing glucose tolerance (Giridhari et al., 2011; Vongsak et al., 2013). AEMOL also improves glucose tolerance in normal, sub, and mild diabetic rats. In positive control, metformin was used as reference drug and interestingly it has found that extract is more effective than reference drug. However, good antidiabetic potential has been seen in severely diabetic rats with damaged islets concluding that, that the AEMOL has some direct effect on tissue utilization of glucose (Gray et al., 2000), absorption of glucose into the muscles and adipose tissues and hepatic gluconeogenesis (Jaiswal et al., 2009).

The results of the *in vitro* α-amylase and α-glucosidase inhibition test displayed that AEMOL had a similar inhibitory effect with lower IC_50_ value in comparison with standard drug acarbose. This *in vitro* finding suggested that AEMOL could decrease the postprandial glucose level by inhibiting the activity of α-amylase and α-glucosidase, which are important enzymes in the digestion of the complex carbohydrates into absorbable monosaccharides in the food. The kinetic parameter, maximal velocity (*V*_max_) is regarded as the glucose uptake rate in the presence as well as in the absence of AEMOL. In the presence of extract, *V*_max_ was decreased in glucose absorption through intestinal membrane indicating that the transmembranal glucose transport was significantly decreased and it was vice versa for glucose absorption in skeletal muscle. However, the *K*_m_ remained unaltered in both the *ex vivo* experiment either supplemented with extract or without extract. This phenomenon signaling that the AEMOL act by bringing a non-competitive type of inhibition of transport of glucose at the level of the small intestine which was due to the inhibition of glucose transporter proteins activity.

This kinetic study only supports the *ex vivo* study and similar type study with other plant extract has also been reported (Therasa et al., 2014). This *ex vivo* study was performed only to provide a biological environment to the extract and to conclude the probable mechanism of extract.

Herbal medicine with complete quality controlled profile is preferable to the regulatory bodies. Previously, antidiabetic activity of *M. oleifera* has been carried out without having an analytical study. Quality control analysis of AEMOL showing antidiabetic potential has been carried out through HPLC. This HPLC data can be used as a quality control parameters of the extract with its antidiabetic potential. Along with HPLC fingerprinting analysis of aqueous extract, gallic acid and rutin were quantitatively analyzed. Antidiabetic properties of AEMOL may be due to the presence of gallic acid, rutin, and other flavonoids and phenolic metabolites (Mbikay, 2012; Dawood and Fathy, 2014). Metabolic profiling with tentative identification of polar and lipophilic metabolites was carried out by GC-MS. A total of 43 polar and 40 lipophilic metabolites were identified in AEMOL. In general, the aqueous extract contains polar metabolites and quite often several fatty acids with other non-polar metabolites were also found due to its solubility and further, these are separated according to partition coefficient. Because of the previous concept, polar and lipophilic metabolites were analyzed by GC-MS. Thus, GC-MS identification is not the real identification and the only basis of retention time, it is being used for quality control analysis because of two different metabolites should have different retention time. HPLC and GC-MS analysis identify the constituents present in aqueous extract which usually polar secondary metabolites such as glycosides, phenols, saponin, and tannins and some primary metabolites such as carbohydrate and proteins. Usually non-polar metabolites such as carotenoids present in ethanolic extract were absent in aqueous extract.

Plant extracts may have several metabolites but all these metabolites may not be active. Some metabolites are in bioactive form, some will be bioactive after its administration, and some are not being bioavailable. GC-MS analysis of both polar and lipophilic metabolites resulted in a complete metabolic signature of AEMOL after its oral administration at a dose of 100 mg/kg with tentative identification. Among, 43 polar and 40 lipophilic metabolites, only 9 polar and 25 lipophilic metabolites were absorbed, thus these are bioavailable. Some additional metabolites which are not present in the extract were found in the blood after its oral administration and this phenomenon may be explained as that these are being overexpressed or may convert from one non-bioavailable metabolite to bioavailable metabolites. Conversion of orally administered metabolites may be due to gut microbiota (Saha et al., 2016). A number of metabolites absorbed in systemic circulation were higher in lipophilic metabolites than polar metabolites.

PCA was applied on identified aqueous soluble metabolites of all the chemotypes. A total of 56.06% of the variance could be explained by these two principal components. This precise clustering of metabolites identified in aqueous extract and in a blood sample collected at different time intervals showed a significant variation. Metabolomics is useful for the chemical and pharmacological standardization of plant extract (Ulrich-Merzenich et al., 2007) and it has the potential to make a revolution in natural product research and to advance the development of scientifically based herbal medicine (Wang et al., 2005). Metabolomes of medicinal plants are particularly a valuable natural resource for the evidence-based development of new phytotherapeutics and nutraceuticals (Shyur and Yang, 2008). From the PCA score plot, it can be seen that similar type of metabolites was found in aqueous extract and in blood collected at different interval except for 1 and 8 h.

**FIGURE 10:**
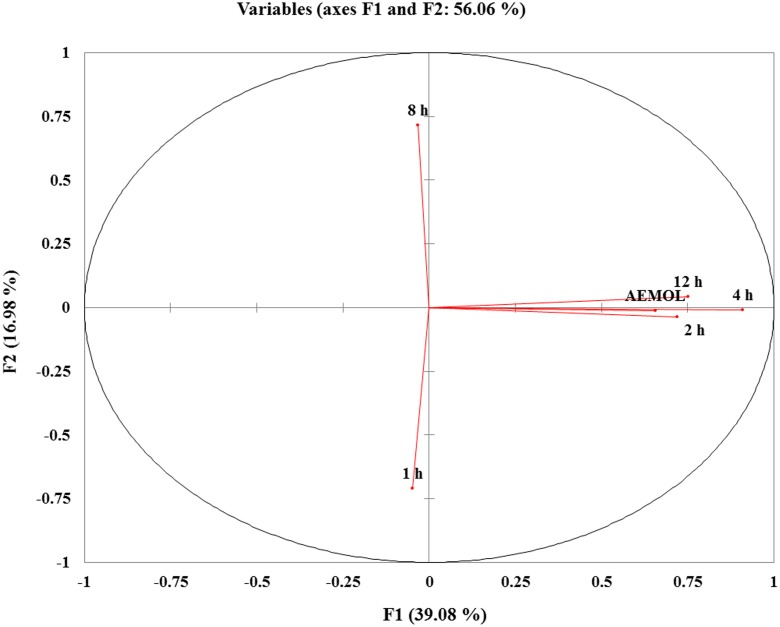
PCA score plots of variable factor map/correlation circles showing the different clusters of samples based on their metabolite abundancy. The sample in same quadrant having similarity in metabolite pattern. Similar metabolite pattern in mother extract and in blood collected at 2, 4, and 12 h after oral administration were found and these are in the same quadrant. While metabolite pattern in 1 and 8 h blood sample have different metabolite patterns.

By reducing the multidimensional data sets, 146 (62 polar and 84 lipophilic metabolites) variables were reduced into two principle components, which were assigned as *x*-axis (PCI) and *y*-axis (PC2) in the analysis. The eigenvalues and the percentage variance and percentage cumulative are shown in **Table 3**. The analyzed data in which variables were centered and scaled to unit variance. The group wise analysis of first principal component (Dim.1/PC1) expressed 39.08% of the variance in the data, and the second principal component (Dim.2/PC2) expressed 16.98% of data variance. A sample of mother extract used as a control was closely clustered with blood sample in PCA plot (**Figure 10**). Furthermore, through prominent clustering indicate that the analytical method for extract was stable and reproducible. A clear separation among the samples collected at different time intervals and in extract has been observed, certifying is a real separation between the groups and also in samples, as the separation is seen despite no class data being included in the algorithm.

**Table 3:** Eigenvalues of metabolite variability in extract and in blood sample.

	AEMOL	1 h	2 h	4 h	8 h	12 h
Eigenvalue	2.345	1.019	0.980	0.909	0.553	0.195
Variability (%)	39.079	16.980	16.341	15.144	9.210	3.247
Cumulative %	39.079	56.059	72.400	87.544	96.753	100.000

**FIGURE 11:**
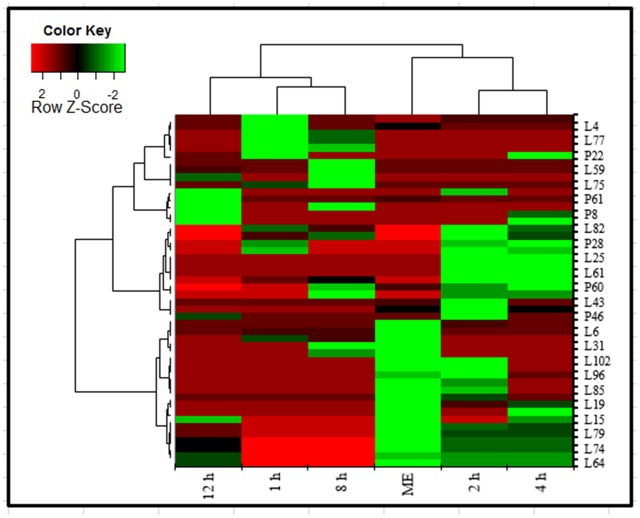
Heatmap analysis of polar and lipophilic metabolites present in aqueous extract and found in blood sample collected at different time intervals after oral administration of aqueous extract in the rat. Metabolites were analyzed by GC-MS. Heatmap represents unsupervised hierarchical clustering of groups (rows). The row displays metabolite and the column represents the samples. Lower abundance of metabolites in samples were displayed in green, while higher abundance metabolites were displayed in red. The brightness of each color corresponded to the magnitude of the difference when compared with the average value. Metabolites were compared with mother extract and metabolites present in the blood after its oral administration. Heatmap visualization constructed based on the differential in metabolites of importance for the bioavailable metabolite in aqueous extract.

The heatmap, executed in XLSTAT tool commonly used for unsupervised clustering, were constructed based on the potential metabolites of importance, which were further extracted with OPLS-DA analysis. Using our metabolomics platform, the statistically important metabolites were studied. PCA was used firstly to investigate general interrelation among aqueous extract and in blood sample based on metabolites abundance, including clustering and outliers among the samples. We specifically introduced a new way to examining metabolite abundance in extract and in a blood sample at different time intervals after its oral administration. The heatmaps, commonly used for unsupervised clustering, were constructed based on the potential candidates of importance, which were extracted with OPLS-DA analysis. A significant separation using heatmap analysis was obtained for the difference between AEMOL vs. blood sample. Using our metabolomics platform, interestingly, the heatmap visualization (**Figure 11**) using in computational systems analysis could be achieved for identification of similarity and dissimilarity of metabolites in the sample. Among 62 polar and 84 lipophilic metabolites in AEMOL and blood sample, metabolites having a higher percentage of contribution were only shown in heatmap. It has been observed that mother extract has devoid of major contributing metabolites which were indicated by green color in heatmap. **Figure 11** showing that less number of lipophilic metabolites and higher abundancy in polar metabolites were found in mother extract. While blood sample having more lipophilic metabolites than polar metabolites. The blood sample with discriminant metabolite abundance was easily distinguished from AEMOL by heatmap approach when using autoscaling methods. To determine whether these metabolites which were identified as bioavailable metabolite using our metabolomics platform, we performed a preliminary validation using a second set of samples which were performed in a similar way.

## Conclusion

The aqueous extract of *M. oleifera* leaf protects pancreas against ROS-mediated damage by enhancing cellular antioxidant defenses and minimizing hyperglycemia in STZ- and HFD-induced diabetes might be due to the enhancement of glucose uptake in skeletal muscle, stimulating insulin secretion, inhibiting of alpha-amylase and alpha-glucosidase enzymes (**Supplementary Figure S1**). These findings support the multiple mechanism of aqueous extract of *M. oleifera* as a potential adjunct dietary treatment for the prevention and/or management of diabetes. The experimental findings clearly indicate an exciting opportunity to develop a potent orally active antidiabetic phytopharmaceuticals from this plant in the future management of diabetes.

## Author Contributions

WK contributed to conceptualization, literature review, experimental studies, data curation, data interpretation, and writing original draft. RP contributed to investigation and manuscript preparation. KC and SP contributed to experimental studies and data curation. SA contributed to conceptualization, investigation, data interpretation, and manuscript critical evaluation.

## Conflict of Interest Statement

The authors declare that the research was conducted in the absence of any commercial or financial relationships that could be construed as a potential conflict of interest. The reviewer PB declared a shared affiliation, with no collaboration, with several of the authors, WK, RP, SA, to the handling Editor.

## Supplementary Material

FIGURE S1 Proposed mechanism of action of AEMOL.

TABLE S1 List of polar metabolites identified by GC-MS analysis.

TABLE S2 List of lipophilic metabolites identified by GC-MS analysis.
