# Supplementary material for: Hypoglycemic Potential of Aqueous Extract of Moringa oleifera Leaf and In Vivo GC-MS Metabolomics
Source: Front Pharmacol. 2017 Sep 12;8:577. doi: 10.3389/fphar.2017.00577 (PMC5601078; doi:10.3389/fphar.2017.00577)
Supplement: FIGURE S1 — Proposed mechanism of action of AEMOL. [file Presentation_1.PPTX]

## Slide 1
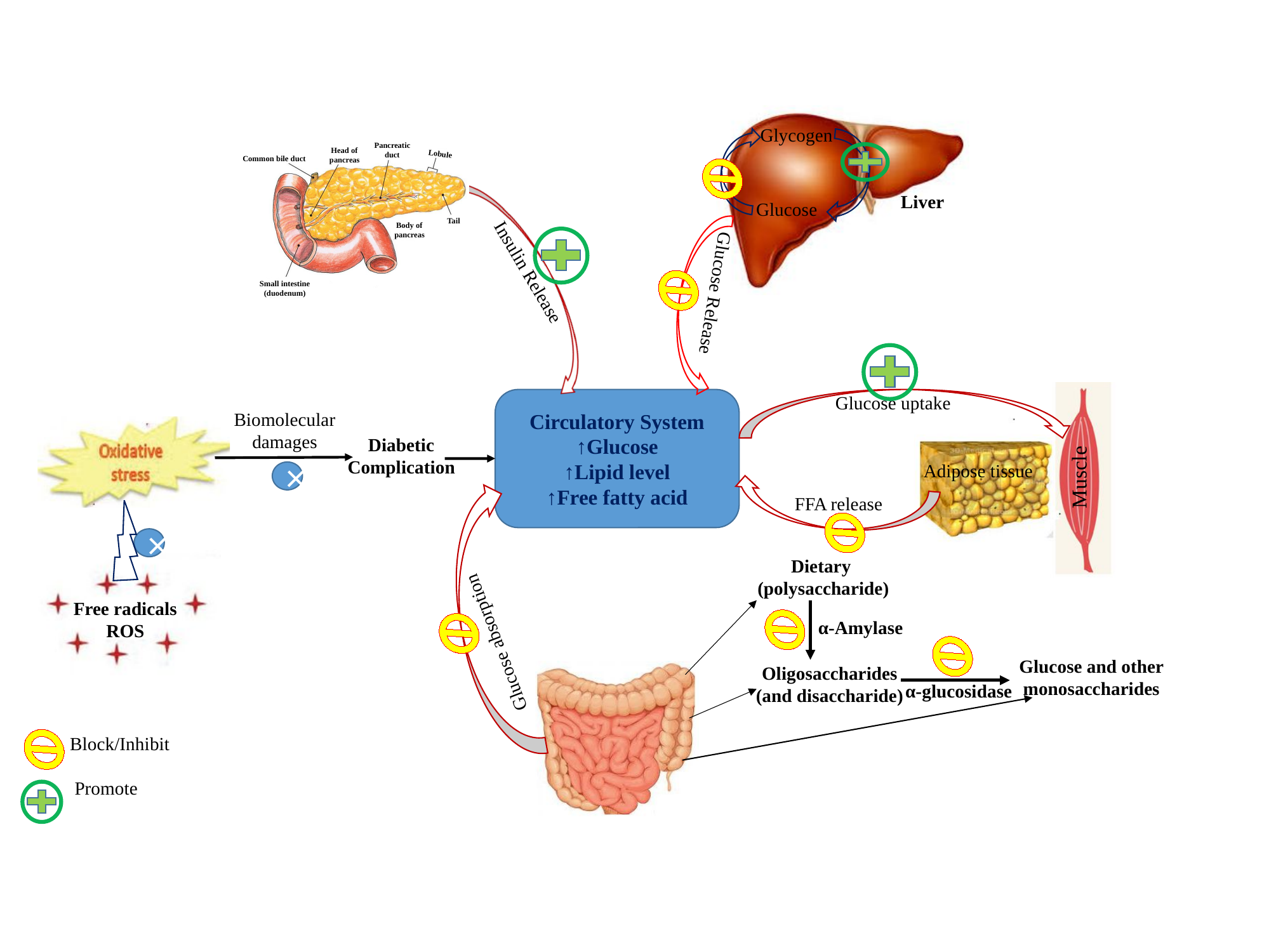

Glycogen
Glucose
Pancreatic duct
Head of pancreas
Lobule
Common bile duct
Tail
Body of pancreas
Small intestine
(duodenum)
Liver
Insulin Release
Glucose Release
Glucose uptake
Circulatory System
↑Glucose
↑Lipid level
↑Free fatty acid
Biomolecular damages
Diabetic Complication
Adipose tissue
Muscle
×
FFA release
×
Free radicals
ROS
Dietary
(polysaccharide)
Glucose absorption
α-Amylase
Glucose and other monosaccharides
Oligosaccharides
(and disaccharide)
α-glucosidase
 Block/Inhibit
 Promote
